# CARE TRAJECTORIES AND WELLBEING OUTCOMES OF OLDER CARERS

**DOI:** 10.1093/geroni/igad104.3097

**Published:** 2023-12-21

**Authors:** Norah Keating, Janet Fast, Choong Kim

**Affiliations:** University of Alberta, Edmonton, Alberta, Canada; University of Alberta, Edmonton, Alberta, Canada; University of Alberta, Edmonton, Alberta, Canada

## Abstract

The UN Decade of Healthy Ageing states that family carers should not be held responsible for care. Yet families continue to provide the majority of care to members with long term health problems and disabilities. Their contributions to the economy remain unrecognized; their costs noted but relegated to private family matters. The purpose of this presentation is to determine wellbeing outcomes of older Canadians with diverse life course trajectories of care. Data are from the 2018 Statistics Canada nationally representative survey on caregiving. Sample for this study is ≈3000 people 65+ with one or more care episodes across their life course. Based on earlier conceptual and empirical work, we recreated 5 care trajectories. Wellbeing was measured based on material, relational and subjective domains. We conducted multivariate analyses (OLS, logistic and ordered logistic regressions) appropriate to the nature of the dependent variable) to assess whether care trajectory type predicted later life wellbeing. Results show significant differences in wellbeing among trajectory types. Serial carers had longest years of care and most care episodes. They experienced the most negative wellbeing outcomes compared to carers with other trajectory types in material wellbeing (poorer physical and mental health, lower employment income), and subjective wellbeing (higher stress). There were no significant differences across care trajectory types in relational wellbeing (loneliness). We discuss the place of public policy in addressing patterns of cumulative disadvantage in life courses of family care; and call for the development of indicators of wellbeing domains that best reflect these outcomes.

